# Misuse of topical over‐the‐counter medication: A case report

**DOI:** 10.1002/ccr3.8949

**Published:** 2024-05-13

**Authors:** Mahesh Mathur, Neha Thakur, Sunil Jaiswal, Nabita Bhattarai, Sambidha Karki, Sandhya Regmi, Supriya Paudel

**Affiliations:** ^1^ Department of Dermatology College of Medical Sciences Bharatpur Nepal

**Keywords:** atrophy, over‐the‐counter medications, topical corticosteroids, vehicles

## Abstract

Overuse of topical medication is a national issue. Over‐the‐counter (OTC) medications are dispensed to patients directly without a physician's prescription and when used improperly without proper knowledge can lead to their misuse and toxicity.

## 1 INTRODUCTION

Overuse of topical medication is recognized globally and is a national issue. Without a physician's prescription, over‐the‐counter (OTC) medications are dispensed to patients directly. These medications when used improperly without proper knowledge and indication lead to their misuse.^1^ Topical OTCs are readily available in pharmacies in our part of world. Non‐labeled indication of topical glucocorticosteroids is rising thus increasing their side effects. Their misuse is expanding among patients and pharmacists, and physicians are themselves responsible for local and systemic consequences. Topical corticosteroids (TC) have anti‐inflammatory, anti‐proliferative, vasoconstrictive, and immunosuppressive action. Side effects due to their misuse lead to telangiectasia, acneiform eruption, hypertrichosis, folliculitis, suppression of the hypothalamic–pituitary–adrenal (HPA) axis, rosacea, skin atrophy, and hypopigmentation.^2^ We hereby present a case of an 18‐year‐old female after misuse of topical medications on her anterior chest.

## 2 CASE PRESENTATION

**FIGURE 1:**
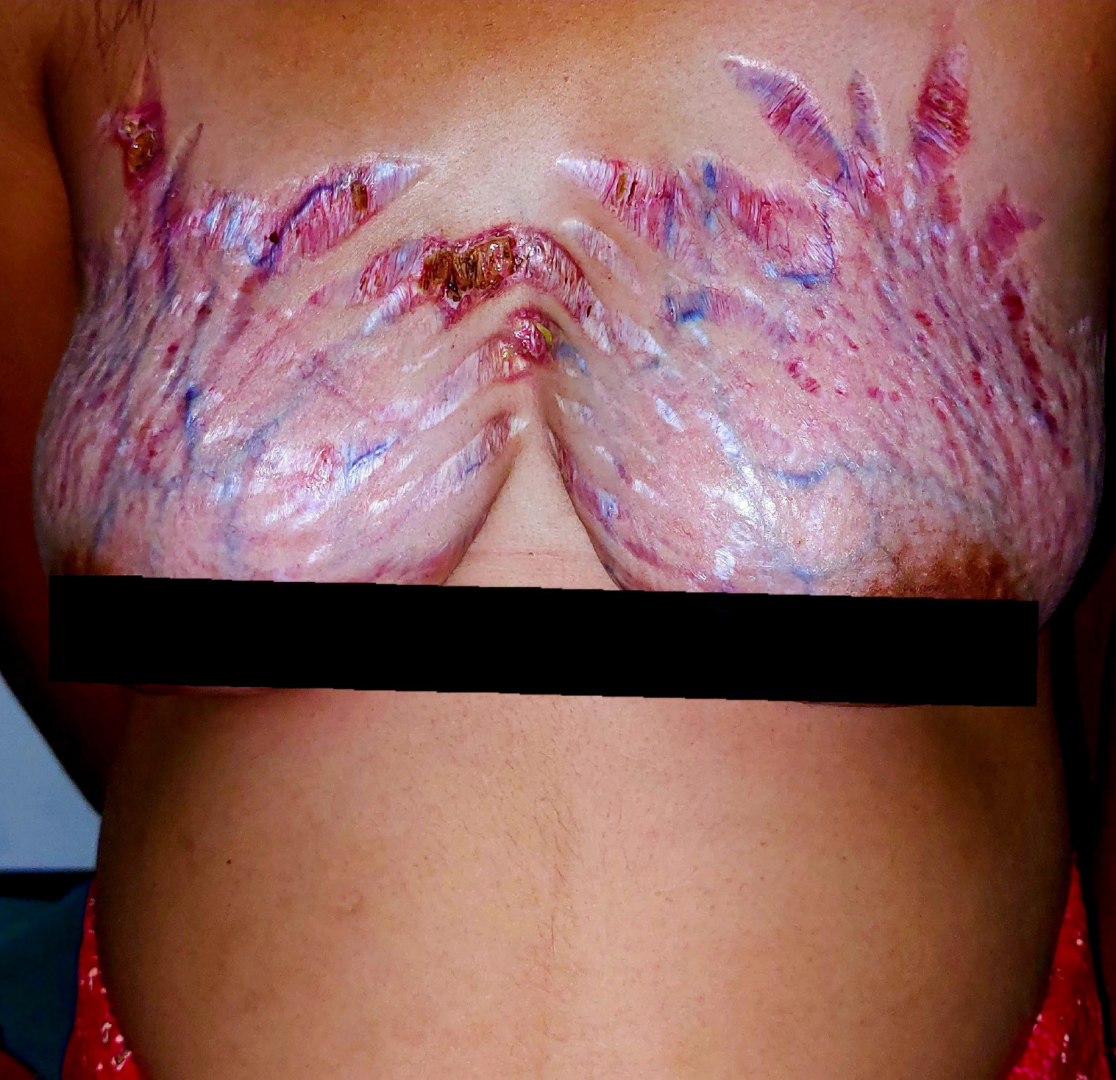
Striae with areas of atrophy, telangiectasias, and visible veins on bilateral breast with crusting and ulceration following prolonged use of Zalim lotion and clobetasol propionate 0.05% in 18‐year‐old pregnant women.

An 18‐year‐old primigravida at 33 weeks of gestation presented to the Dermatology clinic with a 6‐month history of itching and burning sensation on the bilateral breast. On examination, she had shiny atrophied skin with striae, telangiectasias, ulceration, crusting, and prominent veins (Figure 1). This is her first visit to a doctor for the complaints.

On further history taking, she revealed that she developed an itchy and erythematous lesion on her breast 6 months back for which she applied OTC medications on and off. These include phenol, tincture iodine, and crystal violet combination available as Zalim lotion used along with clobetasol propionate 0.05% cream, a superpotent topical corticosteroid.

## 3 METHODS

Routine baseline investigations had normal findings. Therefore, the patient was counseled about the prognosis and educated on prohibition of use of topical medication without a doctor's prescription in the future.

## 4 CONCLUSION AND RESULTS

Irrational combinations of topical glucocorticosteroids and other medications are made by companies and sold by pharmacies. The combination of antibacterial and antifungal agents has led to the emergence of resistant organisms. Abuse and misuse of this unethical combination are common warranting the formulation and implementation of strict laws from the government against unlawful manufacturing and sale of these medications.

## 5 DISCUSSION

The FDA has approved TC use for inflammatory and pruritic dermatosis. Well‐known indications for TC are psoriasis, lichen planus, lichen simplex chronicus, vitiligo, eczema, atopic dermatitis, discoid lupus erythematosus, lichen sclerosus, and also other conditions including melasma, acne, urticaria, and undiagnosed skin rashes.^3^ Vehicles for delivery of TS include ointments, creams, lotions, gel and foams. The most common local side effects due to prolonged treatment include atrophy, striae, rosacea, perioral dermatitis, acne, and purpura. Repeated use of topical corticosteroids causes epidermal thinning, dermal connective tissue changes, reduced collagen and ground substance, and loss of vascular connective tissue support leading to wrinkled, atrophied, and shiny skin along with telangiectasias and visible veins. Atrophy occurs due to persistent use of TC in the same area due to the anti‐mitotic effect. It presents with a burning sensation as seen in our patient. Striae occurs due to injury to the dermis and dermal edema and inflammation result in collagen deposition at the site of mechanical stress. The fatty acid concentration increases after TC use leading to colonization by bacteria and acneiform eruption. Systemic toxicity including glaucoma, HPA axis suppression, Cushing syndrome, hypertension, and hyperglycemia is less as the per‐cutaneous absorption is low but can occur with prolonged use of high‐potent TCs. Fortunately, this was not present in our patient. Stoppage of TC can reverse atrophy but takes several months to normalize and the disfigurement is permanent.^3^, ^4^

Similarly, phenol is the oldest antiseptic and antipruritic aromatic organic compound derived from benzene. It promotes denaturation and coagulation of epidermal keratin proteins, is readily absorbed through the dermis, and has immediate caustic action leading to discomfort, burns, and eventual skin necrosis.^5^ Tinctures of iodine is a solution of iodine 5% plus potassium iodide 10% in water and crystal violet is a triphenylmethane dye with anti‐bacterial, anti‐fungal, and anti‐helminthic properties. These were previously used to treat dermatophytes but their use is obsolete and can cause potential harm.^6^, ^7^, ^8^

TC use in dermatophytosis is a relative contraindication but patients are found applying TC due to easy access as used by our patient. Zalim lotion and topical steroids are two of the commonly misused topical preparations in our part of the world. Public awareness regarding irrational use of drugs is a need of our country. Proper counseling and the concept of a fingertip unit for steroid application are needed as patients may not return for a follow‐up. Dermatologists, pharmacists, nurses, and general medical practitioners should work in collaboration to solve the issue.

## AUTHOR CONTRIBUTIONS

**Mahesh Mathur:** Conceptualization; formal analysis; resources; supervision; validation; visualization; writing – original draft. **Neha Thakur:** Conceptualization; formal analysis; resources; supervision; validation; visualization; writing – original draft. **Sunil Jaiswal:** Formal analysis; resources; supervision; visualization; writing – original draft; writing – review and editing. **Nabita Bhattarai:** Resources; writing – review and editing. **Sambidha Karki:** Resources; writing – review and editing. **Sandhya Regmi:** Resources; writing – review and editing. **Supriya Paudel:** Conceptualization; formal analysis; resources; visualization; writing – original draft; writing – review and editing.

## FUNDING INFORMATION

No funding received for this article.

## CONFLICT OF INTEREST STATEMENT

None.

## CONSENT

Written informed consent was obtained from the patient to publish this report in accordance with the journal's patient consent policy.

## Data Availability

The authors will supply the relevant data in response to reasonable request.
